# A bi-steric mTORC1-selective inhibitor overcomes drug resistance in breast cancer

**DOI:** 10.1038/s41388-023-02737-z

**Published:** 2023-06-01

**Authors:** Delong Meng, Xin Zhao, Yu Chi Yang, Albertas Navickas, Ciara Helland, Hani Goodarzi, Mallika Singh, Sourav Bandyopadhyay

**Affiliations:** 1 Department of Bioengineering and Therapeutic Sciences, University of California San Francisco, San Francisco, CA USA; 2 Department of Biology, Revolution Medicines Inc., Redwood City, CA USA; 3 Department of Biochemistry and Biophysics, University of California, San Francisco, San Francisco, CA 94158 USA; 4 Department of Urology, University of California, San Francisco, San Francisco, CA USA; 5 Helen Diller Family Comprehensive Cancer Center, University of California, San Francisco, San Francisco, CA USA; 6 Bakar Computational Health Sciences Institute, University of California, San Francisco, San Francisco, CA USA; 7 Present Address: Institut Curie, PSL Research University, CNRS UMR3348, INSERM U1278, Orsay, France

**Keywords:** Targeted therapies, Breast cancer, Cancer therapy

## Abstract

Activation of the PI3K-mTOR pathway is central to breast cancer pathogenesis including resistance to many targeted therapies. The mTOR kinase forms two distinct complexes, mTORC1 and mTORC2, and understanding which is required for the survival of malignant cells has been limited by tools to selectively and completely impair either subcomplex. To address this, we used RMC-6272, a bi-steric molecule with a rapamycin-like moiety linked to an mTOR active-site inhibitor that displays >25-fold selectivity for mTORC1 over mTORC2 substrates. Complete suppression of mTORC1 by RMC-6272 causes apoptosis in ER+/HER2− breast cancer cell lines, particularly in those that harbor mutations in *PIK3CA* or *PTEN*, due to inhibition of the rapamycin resistant, mTORC1 substrate 4EBP1 and reduction of the pro-survival protein MCL1. RMC-6272 reduced translation of ribosomal mRNAs, MYC target genes, and components of the CDK4/6 pathway, suggesting enhanced impairment of oncogenic pathways compared to the partial mTORC1 inhibitor everolimus. RMC-6272 maintained efficacy in hormone therapy-resistant acquired cell lines and patient-derived xenografts (PDX), showed increased efficacy in CDK4/6 inhibitor treated acquired resistant cell lines versus their parental counterparts, and was efficacious in a PDX from a patient experiencing resistance to CDK4/6 inhibition. Bi-steric mTORC1-selective inhibition may be effective in overcoming multiple forms of therapy-resistance in ER+ breast cancers.

## Introduction

Breast cancers frequently harbor mutations in the phosphoinositide 3-kinase (PI3K)/mechanistic target of Rapamycin (mTOR) pathway with activating mutations in *PIK3CA* or loss of function mutations in *PTEN*, together occurring in over 70% of cases [1]. In addition, mTOR plays a major role in resistance to various therapies in breast cancer [2–4]. Activation of mTOR resulting in eukaryotic initiation factor 4E (eIF4E) activation confers resistance to tamoxifen [5] and higher expression of mTOR effectors ribosomal protein S6 (RPS6) and eIF4E-binding protein 1 (4EBP1) are associated with poorer survival [6]. mTOR has also been reported to play a role in resistance to cyclin-dependent kinase (CDK) 4/6 inhibitors with CDK4/6 inhibition resulting in activation of mTOR [7]. Despite evidence indicating the importance of this pathway in multiple aspects of breast cancer pathogenesis, the only inhibitors of this pathway in clinical use are the PI3K inhibitor, alpelisib, in PI3K-mutant tumors, and the rapalog everolimus, both approved for use in hormone therapy refractory estrogen receptor positive (ER+) breast cancers. Both have only modest efficacy [8], possibly owing to the fact that both classes of inhibitors induce cell-cycle arrest and are cytostatic rather than cytotoxic to cancer cells [9, 10].

**Fig. 1:**
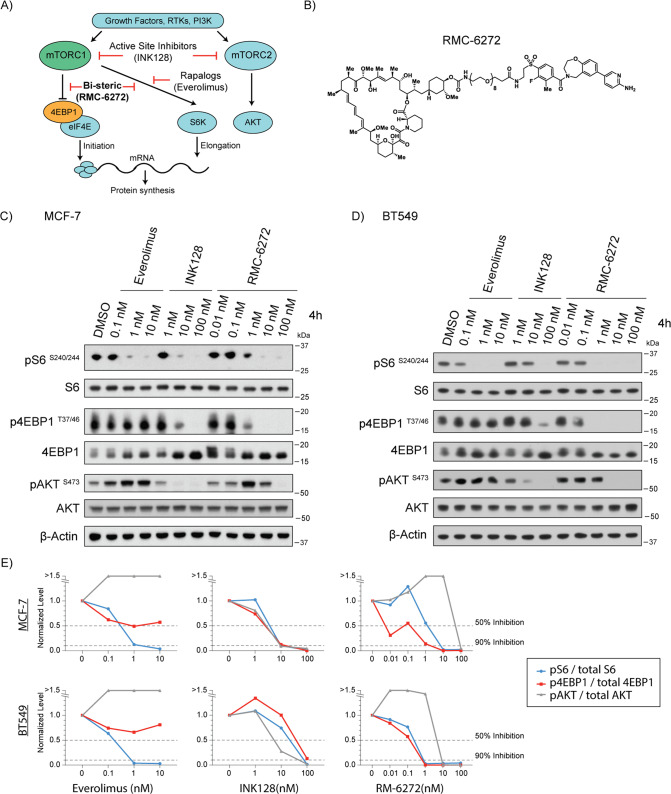
RMC-6272 is a potent and selective mTORC1 inhibitor. **A** Schematic of signaling in the mTOR pathway. An example inhibitor of each class is indicated. RTKs, receptor tyrosine kinases; PI3K, Phosphatidylinositol-3-kinase. **B** Chemical structure of RMC-6272 **C** MCF-7 cells treated with everolimus, INK128 or RMC-6272 for 4 h at indicated doses. Cell lysates were assessed for mTORC1 activity by immunoblotting the phosphorylation of S6 (pS6) at S240/244, the phosphorylation of 4EBP1 (p4EBP1) at T37/46, and for mTORC2 activity by the phosphorylation of AKT (pAKT) at S473. Total S6, 4EBP1, AKT and β-Actin shown as controls. Image represents at least two independent experiments. **D** BT549 breast cancer cells were treated and analyzed as in (**C**). Image represents at least two independent experiments. **E** Quantification of blot intensities in (**C**) and (**D**) indicated phospho-proteins were quantified and normalized to actin and then again to total protein controls. Dotted line indicates 50% and 90% inhibition of signal from baseline.

Targeting this pathway has been a challenge due to the dynamic nature of mTOR and variable effects posed by different means of its inhibition (Fig. 1A). mTOR forms two multiprotein complexes, mTOR complex 1 (mTORC1) and mTOR complex 2 (mTORC2) which are composed of discrete protein binding partners. In response to nutrients and growth factors, mTORC1 regulates mRNA translation and cell size by multiple mechanisms, including phosphorylation of ribosomal S6 kinase (S6K) and eIF4E-binding proteins (4EBPs). First generation rapalogs are mTORC1 specific because they bind to the FRB domain of mTOR that is only accessible on mTORC1, however they only impair substrate phosphorylation of S6K and not other rapamycin-resistant mTORC1 substrates such as 4EBP1 [11]. mTORC2 regulates cell metabolism and proliferation by phosphorylating AGC family kinases such as AKT. mTORC2 has critical functions in the regulation of glucose homeostasis through the regulation of insulin induced glucose uptake. Second generation active-site inhibitors are ATP competitive and impair both mTORC1 and mTORC2 equally, leading to significant toxicity due to hyperglycemia [12]. To overcome limitations of prior molecules, a third generation of mTOR inhibitor was developed in which a rapamycin moiety was joined to an ATP-competitive inhibitor via a chemical linker, exemplified by RapaLink [13]. RapaLink molecules inhibit mTORC1 more potently than rapalogs and other similarly bi-steric variants are highly selective for mTORC1 over mTORC2 [13, 14]. However, these bi-steric molecules induce cytostatic effects in some cell lines suggesting that complete mTORC1 inhibition is associated with different outcomes depending on tumor context and biomarkers for effective treatment with third generation bi-steric compounds are needed.

Here we explore the preclinical applicability of a new bi-steric inhibitor, RMC-6272, representing a class of mTOR inhibitors with over 25-fold selectivity of mTORC1 over mTORC2. Using breast cancer cell lines and xenograft models we show that RMC-6272 causes apoptosis specifically in *PIK3CA* and *PTEN* mutant ER+/HER2- breast cancer cell lines through suppression of the rapamycin resistant, mTORC1 substrate 4EBP1 and reduction of the pro-survival protein MCL1. To test the hypothesis that bi-steric mTORC1-selective inhibition may be effective in overcoming multiple forms of therapy-resistance in ER+ breast cancers, we determined the potential of RMC-6272 in the context of resistance to standard of care therapies in ER+ breast cancer including therapeutics targeting estrogen signaling and CDK4/6 kinases. Our results represent a potential clinical path for the bi-steric mTORC1 inhibitor currently in clinical development.

## Results

### RMC-6272 is a potent and selective bi-steric mTORC1 inhibitor

Bi-steric mTORC1 inhibitors consist of a rapamycin-like core moiety which can allosterically inhibit substrate recognition of mTORC1, and an ATP-mimetic that orthosterically blocks mTORC1 kinase activity [13–16]. RMC-5552 is the first clinical candidate of this class and is being evaluated in a Phase 1 clinical trial (NCT04774952). RMC-6272 is a representative tool compound of this bi-steric class mTORC1-selective inhibitor and shares similar in vitro and in vivo activities with RMC-5552 (Fig. 1B, see “Methods”).

We first assessed the potency and selectivity of RMC-6272 on mTORC1 signaling pathway in the ER+ MCF-7 and triple-negative BT549 breast cancer cell lines (Fig. 1C, D). The rapalog everolimus, active-site inhibitor INK128, or RMC-6272 was added to cells at multiple doses for 4 h. As expected, everolimus inhibited the phosphorylation of S6 at S240/244 downstream of S6 kinase 1 (S6K1) but failed to block the phosphorylation of 4EBP1 at T37/46, both established substrates of mTORC1 [17]. Everolimus did not inhibit phosphorylation of the mTORC2 substrate AKT at S473, consistent with the fact that it blocks substrate accessibility only for mTORC1. INK128 potently inhibited phosphorylation of both mTORC1 substrates (S6 and 4EBP1) and mTORC2 substrate (AKT). The bi-steric mTORC1 inhibitor RMC-6272 potently blocked mTORC1 substrates—both S6 and 4EBP1—at around 1 nM, while it did not inhibit mTORC2 signaling until much higher concentration (10–100 nM) (Fig. 1E). This observation was consistent with our earlier study of similar bi-steric mTORC1 inhibitors in other breast cancer cell lines [14]. In conclusion, the novel bi-steric mTORC1 inhibitor RMC-6272 potently and selectively inhibits mTORC1.

### RMC-6272 causes apoptosis specifically in ER+/HER2-breast cancer cells

**Fig. 2:**
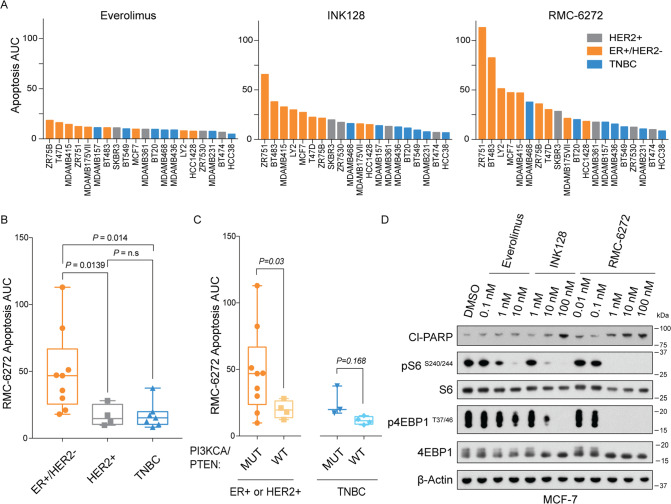
RMC-6272 causes apoptosis specifically in ER+ breast cancer cells. **A** A panel of 20 breast cancer cell lines were treated with Everolimus, INK128 or RMC-6272 for 72 h at multiple doses (1000, 200, 40, 8, 1.6, 0.32, and 0.064 nM) and apoptosis determined by the fraction of YOPRO-1 positively stained cells. Across the same doses, the Area Under Curve (AUC) of this fraction is shown for each line colored by molecular subtypes. Apoptosis AUC upon RMC-6272 treatment partitioned among cell lines with different **B** molecular subtypes and **C** mutation in *PTEN* or *PIK3CA* with ER+ and HER2+ cell lines grouped together. **D** MCF-7 breast cancer cells were treated with Everolimus, INK128 or RMC-6272 for 24 h at indicated doses. Cell lysates were immunoblotted for the apoptosis marker cleaved PARP (Cl-PARP) and the indicated total of phospho-proteins. Image represents at least two independent experiments. Boxes are the 25th to 75th percentiles, the line in the middle of the box is plotted at the median, and whiskers are the 2.5–97.5 percentiles. *P* values are based on Welch’s *t*-test. TNBC triple-negative breast cancer.

To evaluate the preclinical efficacy of RMC-6272 in breast cancer, we tested its effects on cell proliferation and survival in a panel of 20 different breast cancer cell lines spanning the three distinct molecular subtypes: ER+/HER2−, HER2+, and triple-negative breast cancer (TNBC) (Supplementary Table 1). All three generations of mTORC1 inhibitors were able to suppress cell proliferation after 72 h treatment at a series of doses, without obvious differences in sensitivity among the molecular subtypes (Supplementary Fig. 1). We next sought to distinguish the cytostatic from cytotoxic effects by measuring the induction of apoptosis using YOPRO-1 staining after 72 h treatment spanning a relevant range of drug doses. In contrast to everolimus, which is known to be a cytostatic drug and did not induce apoptosis in our panel, both INK128 and RMC-6272 caused dramatically more apoptosis in these cells (Fig. 2A). Interestingly, these two compounds caused apoptosis preferentially in the ER+/HER2- breast cancer cell lines over other molecular subtypes (Fig. 2A, B). Some breast cancers have genetic variation in the oncogenic PI3K/Akt/mTORC1 pathway; therefore, we further stratified the cell lines according to the status of mutations in *PI3KCA* or *PTEN*. We found that among ER+ or HER2+ breast cancer cell lines those carrying mutations in *PI3KCA* or *PTEN* displayed more apoptosis after RMC-6272 treatment (Fig. 2C). To further confirm the apoptosis induction, we treated the ER+/HER2− MCF-7 breast cancer cell line with everolimus, INK128, and RMC-6272 for 24 h, and observed that INK128 and RMC-6272 both induced cleaved poly(ADP-ribose) polymerase (PARP), a molecular marker of apoptosis, whereas everolimus did not (Fig. 2D). Together, the novel bi-steric compound RMC-6272 is a selective mTORC1 inhibitor that causes apoptosis specifically in the ER+/HER2− breast cancers.

### RMC-6272 causes apoptosis through regulating eIF4E and MCL1

**Fig. 3:**
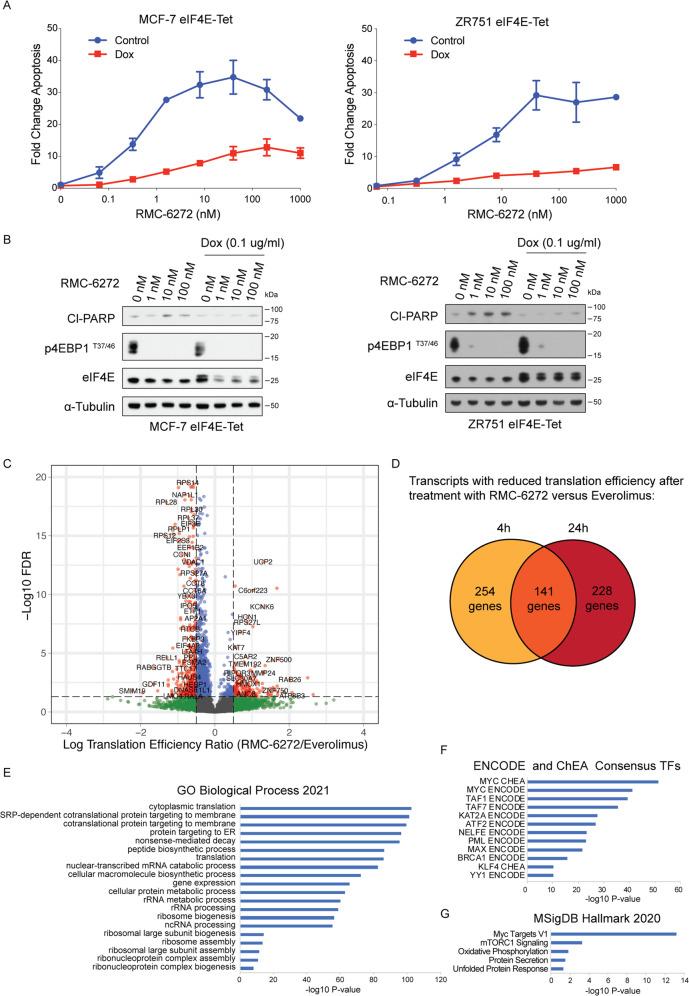
RMC-6272 causes apoptosis through eIF4E mediated repression. **A** MCF-7 or ZR751 breast cancer cells with doxycyclin-inducible eIF4E overexpression were treated with or without doxycycline at 0.1 μg/ml for 72 h to induce the overexpression of eIF4E and then treated with RMC-6272 for 72 h at indicated concentrations. Apoptosis was determined by fraction of total YOPRO-1+ cells and normalized to vehicle. **B** eIF4E inducible MCF-7 or ZR751 cells were treated with doxycycline at 0.1 μg/ml or DMSO as control for 72 h then treated with RMC-6272 at indicated concentration for 24 h, and the lysates were immunoblotted for the apoptosis marker cleaved PARP (Cl-PARP) and indicated molecules. Image represents two independent experiments. **C** Results from ribosome profiling experiment in MCF-7 cells comparing translational efficiencies for individual genes after treatment with 20 nM RMC-6272 versus 20 nM everolimus for 4 h. Cutoff used: |logTER| > 0.5 and FDR < 0.05. **D** Comparison of transcripts with significantly reduced translational efficiencies (based on |logTER| > 0.5 and FDR < 0.05) 4 and 24 h after treatment with RMC-6272 as compared to everolimus. *P* values of enrichment for various groups using gene ontology terms (**E**), consensus transcription factor targets (**F**) hallmark categories in MSigDB (**G**) for the 141 genes in common in (**D**) are shown in bar plots.

We next explored the mechanism of RMC-6272-induced apoptosis in ER+ breast cancer cells by comparing its molecular effects to that of everolimus. mTORC1 phosphorylates 4EBP1 releasing eIF4E to bind mRNAs and initiate 5′ cap dependent RNA translation [18, 19]. Because RMC-6272 specifically inhibits phosphorylation of 4EBP1, we hypothesized that RMC-6272 causes apoptosis through inhibition of 4EBP1 phosphorylation. To genetically reactivate translation suppressed by 4EBP1 inhibition, we stably overexpressed eIF4E in ER+MCF-7 and ZR-75-1 cells using a doxycycline inducible system which reduced apoptosis upon RMC-6272 treatment in both cell lines, assessed by both YOPRO-1 staining and cleaved PARP (Fig. 3A, B). Hence, cell death due to RMC-6272 is a result of inhibition of a rapamycin-resistant function of mTORC1 and independent of mTORC2 inhibition. To understand the global impact of more complete and selective inhibition of mTORC1 we initiated ribosomal profiling experiments comparing mRNA translation after treatment with everolimus versus RMC-6272. We observed a strong reduction in the translational efficiency of 395 transcripts in RMC-6272 treated MCF-7 cells as compared with everolimus treatment after 4 h (Fig. 3C). In total, we identified 141 genes whose translational efficiency was reduced both 4 and 24 h after treatment (Fig. 3D) and pathway analysis indicated more potent reductions in ribosomal genes (Fig. 3E) and MYC target genes (Fig. 3F) incurred by RMC-6272 treatment (Supplementary Table 2). Hence, potent and selective mTORC1 inhibition likely results in a reduction in protein synthesis and oncogenic MYC signaling.

**Fig. 4:**
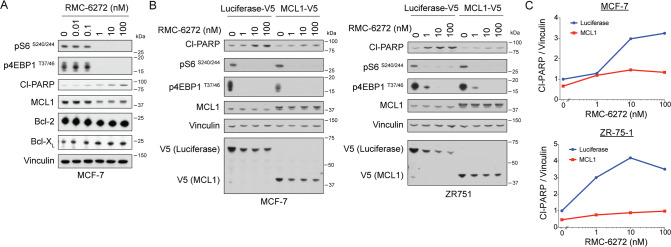
RMC-6272 causes apoptosis through a MCL1 dependent mechanism. **A** Immunoblot of indicated proteins using lysates from MCF-7 cells treated with RMC-6272 at indicated concentration for 24 h. CL-PARP, cleaved PARP. Image represents two independent experiments. **B** MCF-7 or ZR751 cells overexpressing V5-tagged Luciferase (control) or V5-tagged MCL1 treated with RMC-6272 at indicated concentration for 24 h, and the lysates were immunoblotted for indicated proteins. Image represents at least two independent experiments. **C** Quantification of Cl-PARP compared to vinculin loading control in (**B**).

Next, we asked whether RMC-6272 treatment inhibits downstream anti-apoptotic proteins leading to cell death. We did not observe reductions in any known components of the apoptotic pathway in our ribosomal profiling analysis. We assessed the levels of three classical anti-apoptotic proteins—MCL1, Bcl-2, and Bcl-X_L_ (Fig. 4A). Of these three proteins, we only observed a decrease in MCL1 upon RMC-6272 treatment, occurring at similar concentrations that resulted in decreased mTORC1 signaling and increase in cleaved PARP. In addition, RMC-6272 reduced MCL1 expression to a greater extent than everolimus in MCF-7 and ZR751 cells (Supplementary Fig. 2A, B), suggesting that the reduction in MCL1 is a result of enhanced suppression of phosphorylated 4EBP1. To confirm if MCL1 is the key regulator of RMC-6272-induced apoptosis in breast cancer cells, we overexpressed MCL1 in MCF-7 and ZR-75-1 cells, and observed that MCL1 overexpression was sufficient to rescue cells from RMC-6272-induced cleavage of PARP, as compared with the Luciferase control (Fig. 4B, C). Furthermore, the MCL1 inhibitor, S63845, could potentiate apoptosis when combined with a low dose of RMC-6272 (Supplementary Fig. 2C, D), suggesting that MCL1 loss is rate limiting at triggering apoptosis in ER+ breast cancer cells. Together, our data suggested RMC-6272 triggers apoptosis in breast cancers through regulating 4EBP1/eIF4E and downstream protein levels of the key anti-apoptotic protein MCL1.

### Sensitivity is retained after hormone therapy resistance

**Fig. 5:**
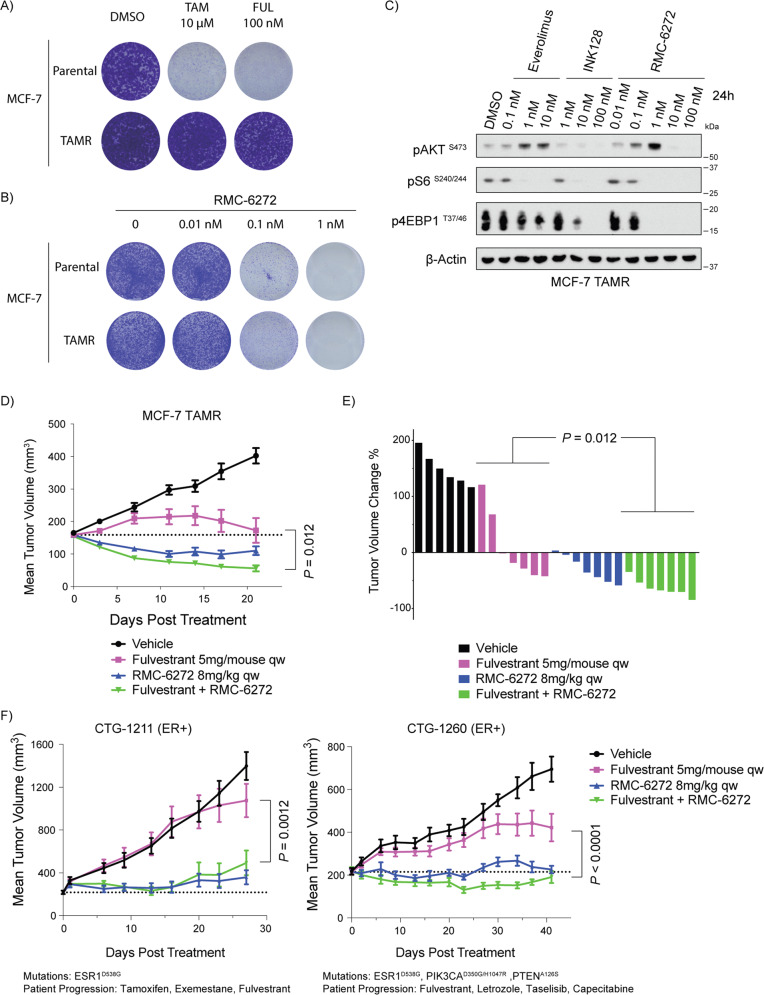
RMC-6272 remains effective in both in vitro and in vivo hormone therapy-resistant breast cancer models. **A** Crystal violet staining of parental or tamoxifen-resistant (TAMR) MCF-7 cells grown in the presence of 10 μM tamoxifen (TAM), 100 nM fulvestrant (FUL), or DMSO as a control. **B** Crystal violet staining of parental or tamoxifen-resistant (TAMR) MCF-7 cells grown in the presence of various concentrations of RMC-6272. **C** Immunoblot of lysates of MCF-7 TAMR cells treated with Everolimus, INK128 or RMC-6272 for 4 h at indicated doses. Image represents at least two independent experiments. **D** Mice carrying MCF-7 TAMR orthotopic xenograft tumors were treated with vehicle, fulvestrant, RMC-6272, or the combination for 21 days. *n* = 7 per group except for vehicle which was *n* = 6. **E** Relative change in tumor volume of individual mice at day 21 colored by treatment group. **F** Single and combination agent dosing in two patient-derived xenograft models derived from ER+ breast cancer patients experiencing acquired resistance to hormone therapies. *n* = 9 mice for each arm in CTG-1211 and *n* = 9 for each arm in CTG-1260 except for vehicle group in CTG-1260 which was *n* = 4 beginning day 9. PI3K pathway mutation and previous patient treatments are indicated. *P* values based on two-tailed Student’s *t*-test.

Advanced ER+ breast cancers are treated with hormone directed therapies and often develop acquired resistance. To test the potential of RMC-6272 in hormone therapy-resistant breast cancers, we utilized a MCF-7 cell line that had been continuously treated with the ER modulator tamoxifen and obtained resistance to 10 µM of tamoxifen and harbor no mutations in *ESR1*, the gene encoding ERα [20]. Tamoxifen-resistant (TAMR) MCF-7 cells are also cross-resistant to the clinically used selective ER degrader, fulvestrant (Fig. 5A), suggesting ER independence. We observed that TAMR MCF-7 cells had comparable sensitivity to RMC-6272 compared with the parental MCF-7 cells (Fig. 5B). Potency and selectivity of mTORC1 inhibition remained the same in TAMR MCF-7 cells as in parental cells (Fig. 5C).

To evaluate RMC-6272’s in vivo efficacy in the hormone therapy-resistant breast cancer models, we orthotopically implanted TAMR MCF-7 cells into the mammary fat pads of mice, and treated the xenografts with fulvestrant, RMC-6272, or the combination for 21 days (Fig. 5D, E). While tumors were partially sensitive to fulvestrant treatment, RMC-6272 induced strong tumor regression during the whole period of treatment as well as at endpoint, and the combination of fulvestrant and RMC-6272 further enhanced the tumor regression effects. We further tested RMC-6272 in patient-derived xenograft (PDX) models derived from patients with ER+ tumors experiencing acquired resistance to hormone directed therapies, CTG-1211 and CTG-1260. Short term pharmacodynamic studies confirmed that RMC-6272 suppressed p-S6 and p4EBP1 in vivo and molecular evidence of apoptosis in the combination was evident after 72 h of treatment with the combination of Fulvestrant and RMC-6272 (Supplementary Fig. 3). CTG-1211 tumors harboring an ESR1^D538G^ mutation associated with hormone therapy resistance [21] were almost completely resistant to fulvestrant treatment, and RMC-6272 alone or in combination with fulvestrant greatly reduced tumor growth rate (Fig. 5F). CTG-1260 tumors harbor both an ESR1^D538G^ mutation and mutations in *PIK3CA* and *PTEN*, and were resistant to fulvestrant in vivo and both single and combination RMC-6272 treatment significantly reduced tumor growth. Hence, hormone therapy-resistant breast cancers are sensitive to RMC-6272 treatment both in vitro and in vivo.

### CDK4/6 inhibitor resistant breast cancer cells are more sensitive to RMC-6272

**Fig. 6:**
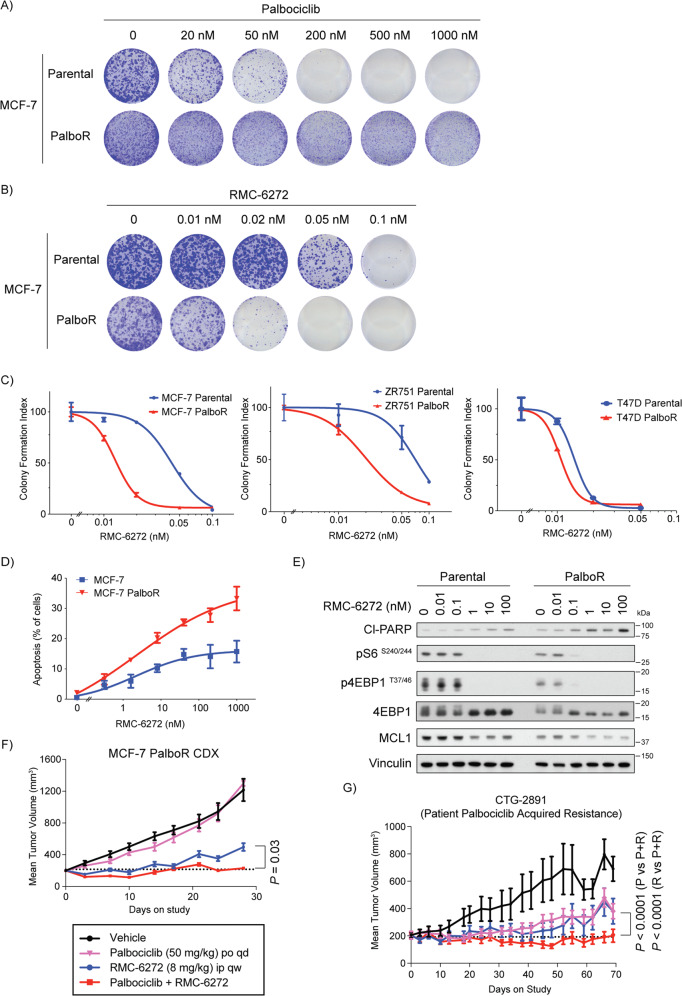
CDK4/6 inhibitor resistant breast cancer cells have increased sensitivity to RMC-6272. Crystal violet staining of parental or Palbociclib-resistant (PalboR) MCF-7 cells grown at a series of concentrations of **A** palbociclib or **B** RMC-6272. **C** Quantification of crystal violet stained MCF-7, ZR752 and T47D parental and PalboR cells in response to various doses of RMC-6272 **D** Fraction of total cells undergoing apoptosis by YO-PRO1 staining in parental or PalboR MCF-7 cells treated with RMC-6272 for 72 h at indicated concentrations. **E** Immunoblot of lysate from MCF-7 parental or PalboR cells treated with the indicated doses of RMC-6272 for 24 h. Image represents at least two independent experiments. Growth of **F** xenografted MCF-7 PalboR cells over 28 days or **G** CTG-2891 PDX model over 70 days treated with the indicated drugs or the combination. Dotted line indicates the 200 m^3^ starting point for treatment. For MCF-7 PalboR xenograft, *n* = 7 mice for vehicle and RMC-6272, *n* = 6 combination, *n* = 5 palbociclib and dosing was 100 mg/kg day 0–3 before dose reduction. For CTG-2891, *n* = 8 palbociclib and RMC-6272, *n* = 7 vehicle and combination. For *P* values based on two-tailed Student’s *t*-test. P palbociclib; R RMC-6272; P + R palbociclib+RMC-6272.

CDK4/6 inhibitors are approved for use in ER+ breast cancers where they significantly increase progression free survival but also suffer from therapy acquired resistance. To test the potential of RMC-6272 in CDK4/6 inhibitor resistant breast cancers, we developed palbociclib-resistant (PalboR) MCF-7, ZR-75-1, and T47D breast cancer cell lines through chronic treatment (Fig. 6A, Supplementary Fig. 4A, B). Growth of PalboR MCF-7, ZR-75-1 and T47D cells were more sensitive to RMC-6272 inhibition as compared with their parental counterparts (Fig. 6B, C, Supplementary Fig. 4C, D). We observed more apoptosis in the PalboR MCF-7 cells upon RMC-6272 treatment compared with parental counterparts via YO-PRO1 staining (Fig. 6D) and increased PARP cleavage after treatment in PalboR MCF-7 and T47D cells compared with parental cells (Fig. 6E, Supplementary Fig. 4E). MCL1 levels were also reduced to a larger extent in PalboR MCF-7 cells than the parental cells after RMC-6272 treatment (Fig. 6E, Supplementary Fig. 4E), suggesting that palbociclib resistance engenders an increase in susceptibility to loss of pro-survival proteins such as MCL1.

Next, we sought to assess the efficacy of RMC-6272 in palbociclib-resistant in vivo models. In a MCF-7 PalboR xenograft model we observed resistance to palbociclib and a marked response to both RMC-6272 alone and in combination with palbociclib, with the combination resulting in significantly stronger tumor growth inhibition (*P* = 0.03, Fig. 6F). We also assessed RMC-6272 in a breast cancer PDX model CTG-2891, taken from a patient with prior treatment and acquired resistance to palbociclib. This model was partially sensitive to palbociclib as well as RMC-6272 as single agent treatment, and the combination of palbociclib and RMC-6272 almost completely blocked tumor growth (Fig. 6G). We did not observe overt toxicity evidenced by changes in mouse body weight throughout this study (Supplementary Fig. 4F). Taken together, these data indicate that RMC-6272 may be an effective therapeutic option to overcome CDK4/6 inhibitor resistance in breast cancer.

## Discussion

The development of next generation bi-steric mTOR inhibitors offer potent and selective inhibition of mTORC1 over mTORC2 and a new approach to target this oncogenic pathway. Previous work has shown that inhibitors in this class do not alter glucose tolerance in mice, a common dose limiting toxicity associated with mTORC2 inhibition [14]. Given the importance of this pathway in many types of cancers, bi-steric inhibitors have been tested in rapamycin-resistant breast cancer cell lines as well as glioma models [13, 22]. To further identify optimal tumor contexts susceptible to bi-steric mTORC1-selective inhibition we focused on breast cancers where this pathway is commonly activated by mutation in upstream regulators such as PIK3CA and PTEN. As opposed to other tumor types such as gliomas which were cytostatic [22], this class of inhibitor caused strong apoptosis selectively in ER+ vs TNBC models and further among those carrying mutations in *PIK3CA* or *PTEN*. Apoptosis in these models was triggered by a selective inhibition of the mTORC1 substrate 4E-BP1 and suppression of the anti-apoptotic factor MCL1, as genetic rescue of EIF4E or MCL1 both suppressed apoptosis induced by RMC-6272. MCL1 has previously been reported as a translational target of mTOR [23], however we did not observe changes in MCL1 translation in our ribosome profiling data and did not observe changes in MCL1 through RNA-seq profiling, hence the cause of the reduction of MCL1 remains unknown and could be potentially due to changes in protein stability as MCL1 is controlled by a number of factors [24].

Advanced ER+ breast cancers are typically treated with hormone therapy and CDK4/6 inhibitors as the standard of care. However, these treatments are often not curative, with progression to acquired resistance occurring in 16 months with fulvestrant [25] and 24 months with the CDK4/6 inhibitor palbociclib [26]. Hormone therapy targets the ER signaling pathway, and several mechanisms have been found to be related to resistance, such as mutations or fusions of ESR1 (the gene that encodes ERα), alterations of ERα coregulations and other genes involved in estrogen signaling, cell-cycle alterations, and epigenetic modifications. Elevated ER expression and transcriptional upregulation of Bcl-2 and c-Myc have also been shown to contribute to anti-estrogen resistance [19]. The interaction between ER signaling and other oncogenic pathways, such as mTORC1 signaling, is also considered to be an important aspect of resistance [27]. For CDK4/6 inhibitors, recent studies have identified potential mechanisms of acquired resistance, including RB1 loss, overexpression of CDK6, and activating alterations in RAS, AURKA, and CCNE2 [28, 29]. Since CDK4/6 partners with Cyclin D1 to promote the phosphorylation of RB1, the amplification of Cyclin E1—which forms a complex with CDK2—has also been reported as a mechanism to bypass the inhibition of CDK4/6 [30].

To understand the role, if any, for a bi-steric mTORC1-selective inhibitor in the context of the current treatment paradigms, we used acquired resistant cell line models of both types of inhibitors and found that RMC-6272 maintained efficacy in these models, and in the case of palbociclib resistance all of our cell line models were more sensitive to RMC-6272 than their parental counterparts. In xenograft models both RMC-6272 alone and in combination with either fulvestrant or palbociclib strongly inhibited tumor growth. Hormone therapy resistance causing mutations in *ESR1* occur in 12–18% of metastatic ER+ breast cancers [31]. RMC-6272 was effective in two ER+ breast cancer PDX models from patients who initially responded but progressed on hormone therapy, and both models harbor ESR1^D538G^, an activating *ESR1* mutation that is associated with hormone resistance [21, 32]. In addition, RMC-6272 was effective alone and in combination with palbociclib in a PDX model taken from a patient who did not respond to palbociclib.

mTORC1 inhibitors have been studied in combination with hormone therapy and/or CDK4/6 inhibitors in melanoma and breast cancer, although the exact mechanisms remain unclear [33, 34]. In ER+ breast cancer, combined inhibition of mTOR and CDK4/6 may result in more complete suppression of E2F-dependent transcription [35] and in our study palbociclib-resistant cell lines demonstrated a loss of RB1 expression and increase of Cyclin E1, both of which can enhance E2F activity and likely contributed to resistance to CDK4/6 inhibition. RMC-6272 mediated mTORC1 inhibition induces apoptosis in palbociclib-resistant cells, potentially through a reduction in total protein synthesis via p4EBP1 inhibition. Future investigation is needed to confirm whether there is a shift in the global mRNA translation in the CDK4/6 inhibitor resistant cells upon RMC-6272 treatment. In addition, it would be of interest to identify potential clinical biomarkers to predict the response to RMC-6272 (or other bi-steric mTORC1-selective inhibitor) treatment. One limitation of our study is that we used immunodeficient mouse models, preventing us from evaluating the potential impact of therapies on immune system and anti-tumor immunity. CDK4/6 inhibitors can promote anti-tumor immunity by increasing the production of type III interferons and tumor antigen presentation and suppressing regulatory T cells, through reducing the activity of E2F [36] and mTOR inhibitors are immunosuppressive [37]. Hence, the immunological effects of RMC-6272 alone and in combination with other treatments in breast cancer warrants more in-depth and comprehensive investigation. In summary, our preclinical data suggest that a bi-steric mTORC1-selective inhibition may be an effective approach for overcoming resistance to standard of care therapeutics in breast cancers, especially in ER+ tumors that harbor mutations that activate mTORC1 including PIK3CA and PTEN. These findings warrant further investigation to confirm the effectiveness of this approach.

## Materials and methods

### Antibodies and chemicals

The following antibodies were purchased from Cell Signaling Technology and used at the indicated dilution for Western blot analysis: phospho-4EBP1 (T37/46; #2855, 1:1000), 4EBP1 (#9644, 1:1000), phospho-S6 (S240/244; #5364, 1:20000), S6 (#2317, 1:1000), phospho-AKT (S473, #4060, 1:1000), AKT-pan (#4691, 1:5000), Cl-PARP (#9541, 1:1000), MCL1 (#94296, 1:2000), β-Actin (#3700, 1:100,000). These antibodies were obtained from Santa Cruz Biotechnology: Vinculin (sc-55465, 1:1000), Bcl-2 (sc-7382, 1:1000), V5 (sc-271944, 1:1000), and α-Tubulin (sc-8035, 1:1000). Bcl-XL (BD-556361, 1:1000) and eIF4E (BD-610269, 1:5000) antibodies were obtained from BD Biosciences. Horseradish peroxidase (HRP) linked secondary antibodies were from Cell Signaling Technology (#7076 anti-mouse or #7074 anti-rabbit, 1:4000). 4-Hydroxy-tamoxifen was purchased from SIGMA-ALDRICH INC (St. Louis, MO). Everolimus and INK128 were obtained from Revolution Medicine (South San Francisco, CA). Fulvestrant (#HY-13636/CS-1267) was purchased from MedChemExpress. MCL1-inhibitor S63845 was from MedChemExpress (#S63845).

The synthesis and characterization of RMC-6272 and RMC-5552 have been described elsewhere [38]. Both as bi-steric inhibitors in which the ether chemical handle was exchanged for a C40 carbamate to enable synthetic tractability and incorporated the XL388-derived (RMC-6272) and MLN0128-derived (RMC-5552) active-site inhibitors, respectively. Based on cellular assays using MDA-MB-468 cells, RMC-6272 and RMC-5552 display very potent inhibition of p4EBP1 (IC50: 0.44 nM and 0.48 M, respectively) and pS6K (IC50: 0.14 nM for both). Both compounds demonstrate selectivity for mTORC1 over mTORC2 (calculated as pAKT IC50/p4EBP1 IC50), with approximately 27-fold and 40-fold selectivity for RMC-6272 and RMC-5552, respectively.

### Cell lines and tissue culture

BT20, BT474, BT549, HCC1428, HCC38, LY2, MCF-7, MDA-MB-231, T47D and ZR75B breast cancer cell lines were purchased from the American Type Culture Collection (ATCC). BT483, MDA-MB-157, MDA-MB-175VII, MDA-MB-361, MDA-MB-415, MDA-MB-436, MDA-MB-468, SKBR3, ZR-75-1 and ZR-75-30 breast cancer cells were purchased from the UCSF Cell Culture Facility (UCSF CCF). Tamoxifen-resistant (TAMR) MCF-7 (10 μM tamoxifen) cells were obtained from Dr. Pamela Munster (UCSF, San Francisco, CA). Palbociclib-resistant (PalboR) MCF-7 (1 μM palbociclib), ZR-75-1 (500 nM palbociclib), and T47D (100 nM palbociclib) cell lines were generated in the lab by gradually increase the concentration of palbociclib in the media. All resistant lines were grown and maintained in the presence indicated doses of the drug. Cell lines were confirmed by STR validation to ensure the authenticity of cell lines. No presence of Mycoplasma was found according to MycoAlert^TM^ Mycoplasma Detection Kit from Lonza Group. Cell lines were grown according to the protocols in Supplementary Table 1.

### Viral transduction and stable cell lines

For stable expression of eIF4E, the pLenti-MCV-Tet-eIF4E-flag plasmid was a gift from Dr. Davide Ruggero’s laboratory. Briefly, HEK293T cells were transfected with pLenti-MCV-Tet-eIF4E-flag together with packaging plasmid VSVG, Gag/pol and Rev. Virus particles were collected after 48 h transfection, filtered through 0.45-mm pore size Millipore filters. Filtered lentiviral supernatant was added to MCF-7 and ZR-75-1 cell lines with polybrene (8 µg/mL). After 72 h incubation, cells were selected in puromycin (2 mg/mL) for 72 h. To induce the expression of eIF4E-flag, cells were treated with doxycycline at indicated concentration.

For MCL1 overexpression, pLX304-MCL1-V5 or pLX304-Luciferase-V5 (a gift from Kevin Janes; Addgene plasmid # 98580) as a control were used to transfect HEK293T cells together with packaging plasmid VSVG, Gag/pol and Rev. Viruses were collected 48–72 h post transfection and concentrated using Lenti-X concentrator (Takara #631231) according to manufacturer’s instructions. MCF-7 cells were infected with viruses with polybrene (8 μg/mL) and blasticidin (10–20 mg/mL) was added 72 h after infection until non-infected cells all died.

### Western blot

Cells were rinsed with PBS and lysed using the Laemmli sample buffer (50 mM Tris pH 6.8, 2% SDS, 0.025% Bromophenol Blue, 10% glycerol, 5% BME). Lysates were boiled for 20 min, resolved using NuPAGE 4–12% SDS–PAGE gels (Life Technologies) and transferred to PVDF membranes (Millipore). Membranes were blocked using the Superblock T20 (TBS) Blocking Buffer (#37536 from Thermo Fisher Scientific), probed with primary antibodies overnight at 4 °C, and horseradish peroxidase (HRP) conjugated secondary antibodies at room temperature for 1 h. The immune complexes were detected by SuperSignal^TM^ West Dura Substrate (#34075 from Thermo Fisher Scientific).

### Proliferation and apoptosis assays

Cells were seeded with their specified medium in 384-well plates (for slow growing cells: 2000 cells/well; for the normal growing cells: 1000 cell/well; for fast growing cells: 500 cells/well) overnight, and then treated with drugs for 72 h. Cell proliferation and cell death was measured by staining with Hoechst (Life Technologies #H3570) nuclear dye at 5 μg/ml and YOPRO-1 (Life Technologies #Y3603) at 500 nM, respectively, and analyzed using a Thermo CellInsight High Content microscope. The raw measurements from each treated well were normalized to the median of vehicle-treated control wells from the corresponding plate.

### Clonogenic assay

Cells were seeded in 12-well microplates overnight at various density according to cell growth rate (for slow growing cells: ~10,000 cells/well; for the normal growing cells: ~6000 cells/well; for fast growing cells: ~3000 cells/well). For PalboR ZR-75-1 cells, due to decreased attachments of the cells to the plates, plates were treated with 5 μg/ml fibronectin (Sigma #F4759) prior to cell seeding. Cells then were treated with drugs at a series of concentrations or DMSO as a control, and media were replaced every 3–4 days. Cells were exposed to drugs or DMSO for about two weeks in total until the DMSO control wells reach confluence.

At harvest, cells were washed with PBS, fixed with cold methanol for 10 min and stained with 0.5% crystal violet for at least 1 h. Plates were rinsed with water and left for drying at room temperature. Pictures of stained colonies were taken using an EPSON Perfection V600 scanner on the following day. For quantification, stains were extracted using extraction buffer (0.1% SDS in 50% ethanol) and absorbance measured by a BioTek plate reader at 562 nm.

### Ribosome profiling

Ribosome profiling was performed based on previous protocol [39]. To process the sequencing data, the ribosome profiling reads were first trimmed using cutadapt (v2.3) to remove the linker sequence AGATCGGAAGAGCAC. The fastx_barcode_splitter script from the Fastx toolkit was then used to split the samples based on their barcodes. Since the reads contain unique molecular identifiers (UMIs), they were collapsed to retain only unique reads. The UMIs were then removed from the beginning and end of each read (2 and 5 Ns, respectively) and appended to the name of each read using umitools (v0.3.3). Bowtie2 (v2.3.5) was then used to remove reads that map to ribosomal RNAs and tRNAs, and the remainder of reads were then aligned to mRNAs (we used the isoform with the longest coding sequence for each gene as the representative). Subsequent to alignment, umitools was used to deduplicate reads. The RNA-seq reads were directly aligned to the same mRNA reference using bowtie2. The translation efficiencies, i.e. the ratio between the RPF and RNA read counts per gene, were compared using Ribolog [40].

### Mouse xenograft studies

Animal studies were conducted in compliance with the regulations of UCSF Institutional Animal Care and Use Committee (IACUC). MCF-7 TAMR cells were orthotopically implanted into the mammary fat pads of female NSG mice. Each mouse was injected with 5 million cells in the cleared mammary fat pads bearing slow release estrogen pellets and allowed to reach a tumor volume of around 150 mm^3^. The xenografts were then randomized into 4 cohort groups (*n* = 8) and treated with vehicle, fulvestrant, RMC-6272, or the combination of fulvestrant and RMC-6272. Fulvestrant was prepared in peanut oil at 25 mg/ml and subcutaneously injected 5 mg/mouse weekly, and RMC-6272 was prepared in 5:5:90 (v/w/v) Transcutol/Solutol HS 15/water and intraperitoneally injected 8 mg/kg weekly. Vehicle was a combination treatment of diluents delivered subcutaneously or intraperitoneally. Tumor volumes were measured every 3–4 days by caliper measurements.

MCF-7 PalboR model was developed by WuXi AppTec Co., Ltd (Suzhou, China). The MCF-7 PalboR tumors were derived in vivo from mice bearing MCF-7 tumors treated with palbociclib daily until resistant growth observed, and resistant tumors were serially implanted into next round of mice continuously treated with palbociclib. The resistant tumors (from passage 4) were implanted into 2–4 donor mice with palbociclib treatment, and when reached 500–1000 mm^3^, the tumors were removed and cut into fragments of ~30 mm^3^, which were then implanted into study mice (Balb/c nude female mice 6–8 weeks old) supplemented with subcutaneous injection of estrogen solution (0.04 mg/mouse) twice weekly since the day of implantation. Mice were randomized into treatment groups with mean tumor volume of ~200 mm^3^ and enrolled into study.

PDX models CTG-1211, CTG-1260, and CTG-2891 were done by Champions Oncology, Inc. (Rockville, MD). Athymic Nude-Foxn1nu female mice 6–8 weeks old were implanted subcutaneously with fragments from PDX tumors. When tumors reached ~150–300 mm^3^, mice were randomized into treatment groups and enrolled into studies.

### Statistical analyses

Data are expressed as averages, with the standard error of mean (±SEM) indicated. Statistical analyses were performed in Graphpad Prism 7 software using unpaired *t* test with Welch’s correction unless otherwise stated in figure legends. Results were considered statistically significant for *P* < 0.05.

## Supplementary information

Supplementary Figures

Supplemental Table 1

Supplemental Table 2

## Data Availability

The data generated in this study are publicly available in Gene Expression Omnibus (GEO) at GSE230627.
