# Supplementary material for: A bi-steric mTORC1-selective inhibitor overcomes drug resistance in breast cancer
Source: Oncogene. 2023 Jun 1;42(28):2207–17. doi: 10.1038/s41388-023-02737-z (PMC10328828; doi:10.1038/s41388-023-02737-z)
Supplement: Supplementary file 1 — Supplementary Figures [file 41388_2023_2737_MOESM1_ESM.docx]

**SUPPLEMENTARY TABLES**

**Supplementary Table 1. Annotation of cell lines used in this study.**

**Supplementary Table 2. Table of gene enrichment analyses of ribosomal profiling data.** Included are different enrichment categories and summary used to generate Figure 3E,F.

**SUPPLEMENTARY FIGURES**

**
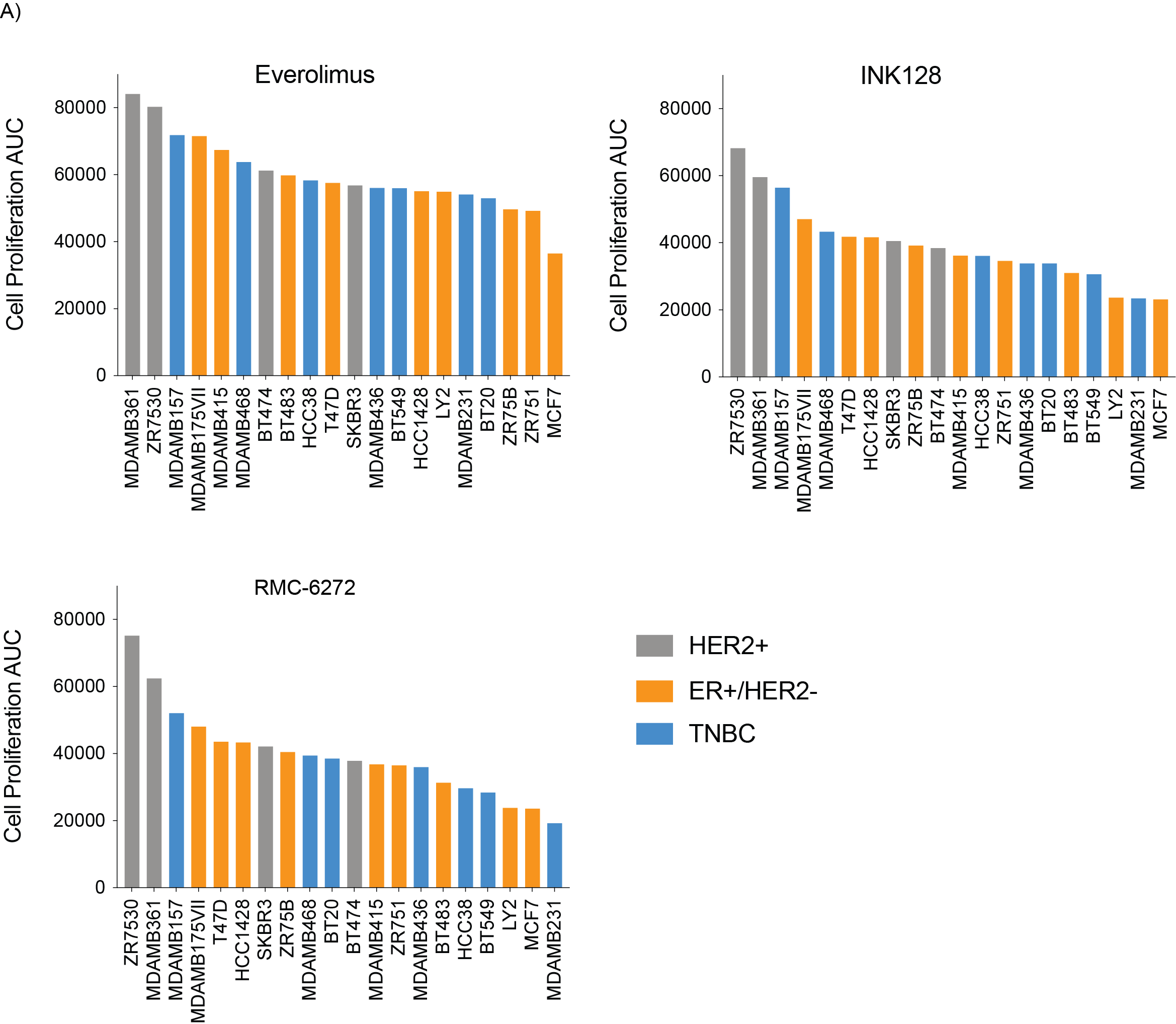
Supplemental Figure 1. mTORC1 inhibitors prevent cell proliferation in breast cancer cells. (A)** A panel of 20 breast cancer cell lines were treated with Everolimus, INK128 or RMC-6272 at multiple doses for 72 hours, and cell number was obtained by Hoechst staining. The Area Under Curve (AUC) of the cell proliferation curve was shown using waterfall plots for these cell lines colored by their molecular subtypes: ER+/HER2-, HER2+, and triple negative breast cancer (TNBC).

**
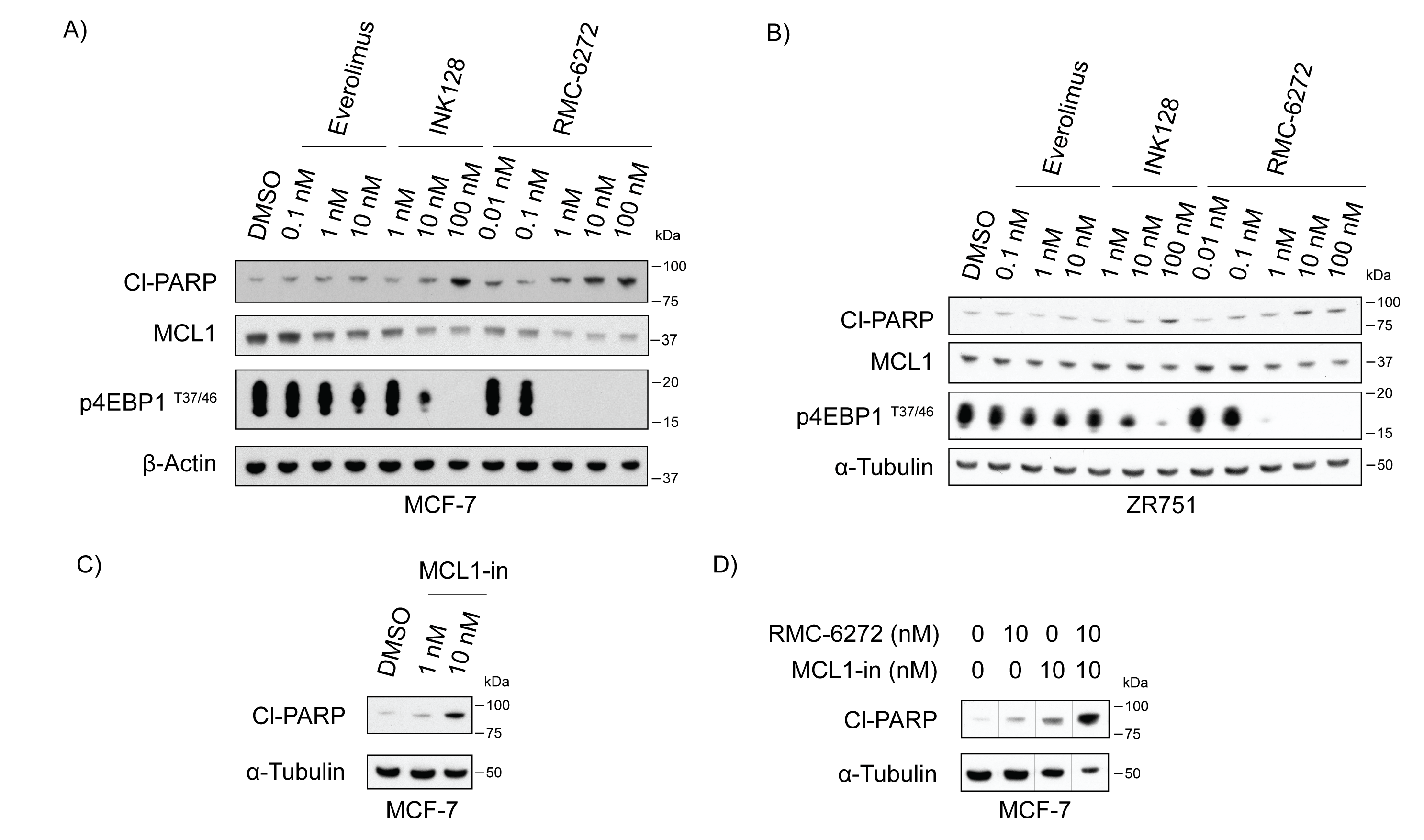
**

**Supplemental Figure 2. RMC-6272 regulates MCL1 expression and MCL1 is a critical regulator of apoptosis in breast cancer cells. (A)** MCF-7 breast cancer cells were treated with Everolimus, INK128 or RMC-6272 for 24 hours at indicated doses. Cell lysates were immunoblotted for the apoptosis marker cleaved PARP (Cl-PARP), anti-apoptotic protein MCL1, the phosphorylation of 4EBP1 (p4EBP1) at T37/46, and β-Actin as a loading control. Samples and some blots are the same with that in Figure 2D. **(B)** ZR751 cells were treated and analyzed similarly as in (A). α-Tubulin was used as a loading control. **(C)** MCF-7 cells were treated with MCL1 inhibitor S63845 (MCL1-in) at 1 nM or 10 nM for 24 hours. Lysates were immunoblotted for Cl-PARP and α-Tubulin. **(D)** MCF-7 cells were treated with either RMC-6272 or MCL1 inhibitor S63845 (MCL1-in) alone at 10 nM, or with a combination of RMC-6272 and MCL1-in for 24 hours. Lysates were immunoblotted for Cl-PARP and α-Tubulin.


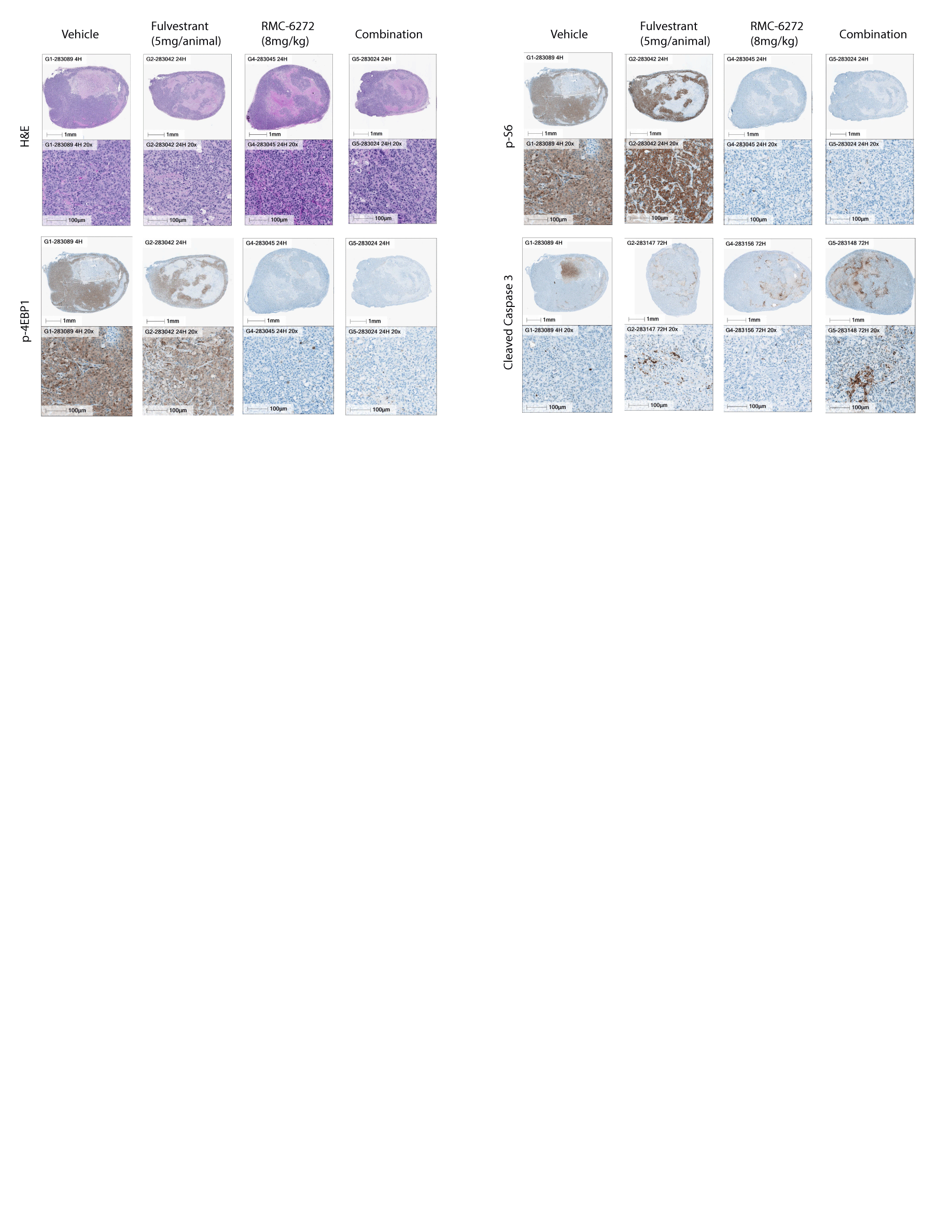


**Supplemental Figure 3. Pharmacodynamic studies in the CTG-1260 PDX model.** Tissue sections taken from tumors harvested from CTG-1260 carrying mice taken at the indicated time points after treatment (4-72 hours). Samples stained with the indicated antibodies or hematoxylin and eosin stain (H&E).


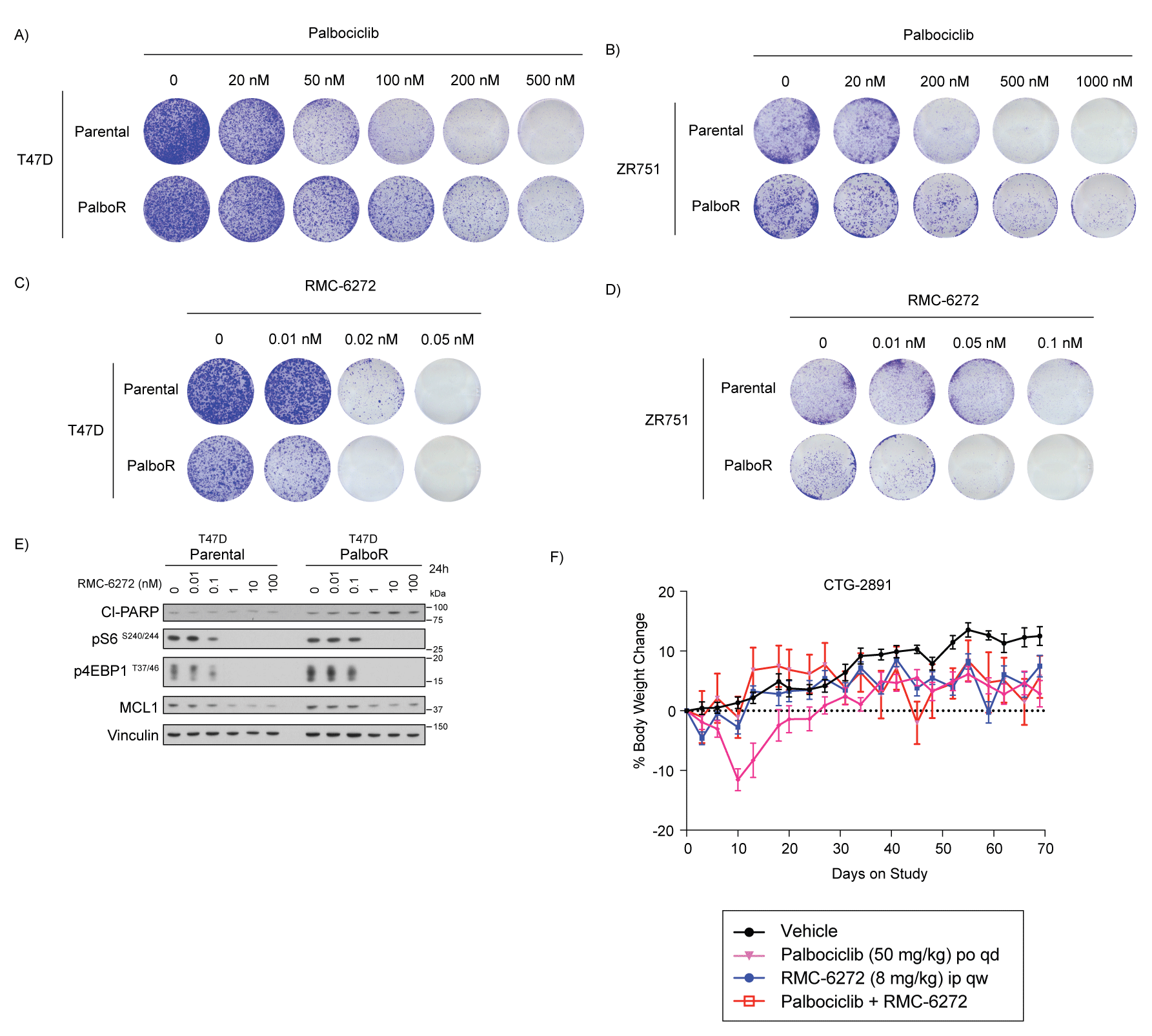


**Supplemental Figure 4. CDK4/6 inhibitor resistant breast cancer cells have increased sensitivity to RMC-6272.** Crystal violet staining of growth of parental or Palbociclib resistant (PalboR) **(A)** T47D cells or **(B)** ZR751 cells in the presence of Palbociclib at a series of concentrations. Crystal violet staining of growth of parental or Palbociclib resistant (PalboR) **(C)** T47D cells or **(D)** ZR751 cells in the presence of RMC-6272 at a series of concentrations. **(E)** Parental or PalboR T47D cells were treated with RMC-6272 for 24 hours at indicated doses. Cell lysates were immunoblotted using indicated antibodies. **(F)** Body weight change of the CTG-2891 PDX model over 70 days of treatment with the indicated drugs or the combination.
